# A survey of education and confidence level among graduating anesthesia residents with regard to selected peripheral nerve blocks

**DOI:** 10.1186/1471-2253-13-16

**Published:** 2013-07-17

**Authors:** Tiffany Sun Moon, Eunjung Lim, Sakura Kinjo

**Affiliations:** 1 Department of Anesthesiology and Pain Management, University of Texas Southwestern Medical Center, Dallas, TX, USA; 2 School of Medicine, University of Hawaii, Honolulu, Hawaii, USA; 3 Department of Anesthesia and Perioperative Care, University of California, San Francisco, CA, USA

**Keywords:** Peripheral nerve block, Ultrasound, Nerve stimulator, Residency

## Abstract

**Background:**

As peripheral nerve blockade has increased significantly over the past decade, resident education and exposure to peripheral nerve blocks has also increased. This survey assessed the levels of exposure and confidence that graduating residents have with performing selected peripheral nerve blocks.

**Methods:**

All program directors of ACGME-accredited anesthesiology programs in the USA were asked to distribute an online survey to their graduating residents. Information was gathered on the number and types of nerve blocks performed, technique(s) utilized, perceived comfort level in performing nerve blocks, perceived quality of regional anesthesia teaching during residency, and suggested areas for improvement.

**Results:**

One hundred and seven residents completed the survey. The majority completed more than 60 nerve blocks. Femoral and interscalene blocks were performed most frequently, with 59% and 41% of residents performing more than 20 of each procedure, respectively. The least-performed block was the lumber plexus block, with just 9% performing 20 or more blocks. Most residents reported feeling “very” to “somewhat” comfortable performing the surveyed blocks, with the exception of the lumber plexus block, where 64% were “not comfortable.” Overall, 78% of residents were “mostly” to “very satisfied” with the quality of education received during residency.

**Conclusions:**

Most of the respondents fulfilled the ACGME requirement and expressed satisfaction with the peripheral nerve block education received during residency. However, the ACGME requirement for 40 nerve blocks may not be adequate for some residents to feel comfortable in performing a full range of blocks upon graduation. Many residents felt that curriculums incorporating simulator training and didactic lectures would be the most helpful method of improving the quality of their education pertaining to peripheral nerve blocks.

## Background

Peripheral nerve blocks (PNBs) have gained popularity over the past fifteen years due to advantages such as good postoperative pain control and the sparing of opioids and their side effects [1]. In addition, peripheral nerve blockade may result in reduced overall cost, a shorter length of stay in the recovery room, and a shorter hospitalization [2]. Improvements in technology, such as better ultrasound equipment and echogenic stimulator needles, have made performing PNBs easier and safer [1]. When compared to general anesthesia, peripheral nerve blocks are advantageous due to avoidance of opioid-related side effects (e.g., nausea/vomiting, urinary retention, pruritus), sparing of the contralateral limb, and decreased incidence of hypotension [2]. Due to these advantages, patients and surgeons are increasingly requesting peripheral nerve blocks.

The Accreditation Council for Graduate Medical Education (ACGME) mandates that residents graduating from all American anesthesiology programs must have performed 40 PNBs upon completion of their residency [6]. Many have argued that this number is not adequate to ensure minimal competence in performing the majority of standard nerve blocks frequently encountered in clinical practice [3]. In addition, there is little information available regarding the differences in the total number of nerve blocks performed, technique(s) utilized (ultrasound [US] guided vs. nerve stimulator [NS] guided) and perceived deficits in training amongst the different programs. A survey conducted by Smith et al. in 1999 found that over 60% of graduating anesthesia residents were not comfortable performing peripheral nerve blocks independently upon completion of residency [4].

Training programs should be accountable to regulatory bodies, and to society, for training physicians who meet the minimum acceptable practice standards. Programs that have deficiencies have been encouraged to augment their curriculums so that residents receive more exposure and education on performing peripheral nerve blocks [5]. In this nationwide survey, we sought to assess graduating residents’ experiences with selected peripheral nerve blocks, perceived comfort level in performing these nerve blocks, perceived quality of regional anesthesia instruction during residency, and suggested areas for improvement.

## Methods

After obtaining exemption from the University of California, San Francisco Committee on Human Research, we compiled a short online survey to assess the level of exposure to PNBs and perceived competency of graduating anesthesia residents in accredited anesthesiology programs in the United States. In June 2011, a list of residency program directors’ emails was obtained from the ACGME website [6] and individual emails were sent to the program directors asking for their program’s participation. The survey was created using the online tool SurveyMonkey®, at http://www.surveymonkey.com. A secure link to the survey was supplied for forwarding to the third clinical anesthesia (CA-3) year residents by each program director. A short narrative was provided explaining the purpose of the survey. It was made clear that participation was completely voluntary and would not be rewarded. The survey was to be completed online by each individual trainee. It would be anonymous and was not tied to the residency programs. A reminder email was sent to the program directors two weeks after the original emails were sent.

The assessed PNBs included interscalene, axillary, femoral, sciatic, popliteal, and lumbar plexus. These nerve blocks were chosen because they encompassed frequently-performed blocks in both the upper and lower extremities. The levels of perceived confidence choices ranged from “not comfortable” to “somewhat comfortable” to “very comfortable.” The survey also asked for each resident’s level of satisfaction with the education they received during residency and which factors they would have deemed most helpful in improving their education in peripheral nerve blockade.

### Statistical analysis

Descriptive statistics (mean, standard deviation, and percentages) were computed to describe the sample characteristics and depict residents’ exposure to various types of PNB blocks, as well as their confidence and satisfaction level. Spearman rank correlations determined the degree of association between number of blocks and level of confidence to each block or overall satisfaction level. All the analyses were conducted in SAS 9.2 (SAS Institute, Inc., Cary, NC) and a P-value <0.05 was considered as significant.

## Results

**Figure 1:**
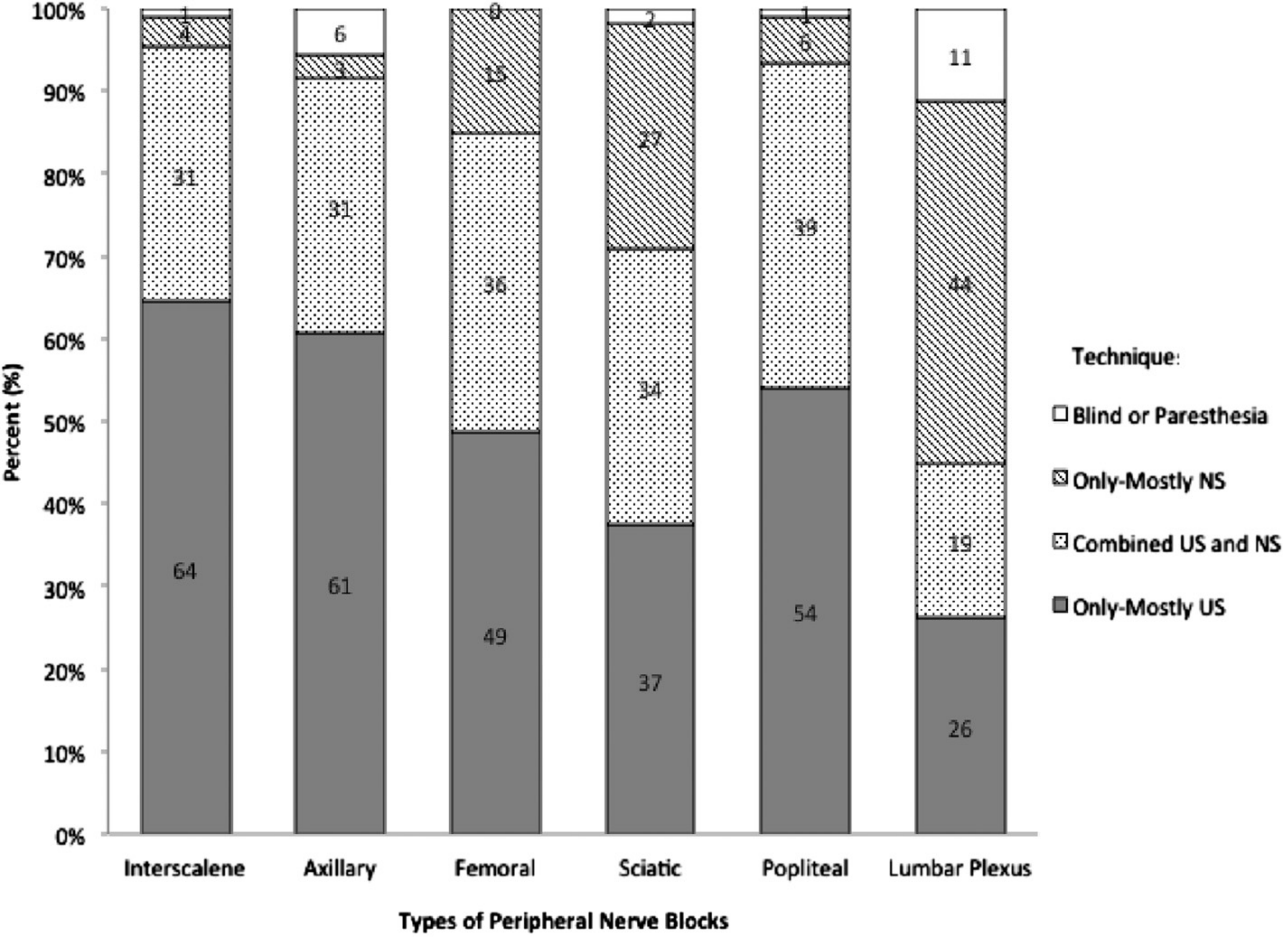
Techniques used for performing selected peripheral nerve blocks.

**Figure 2:**
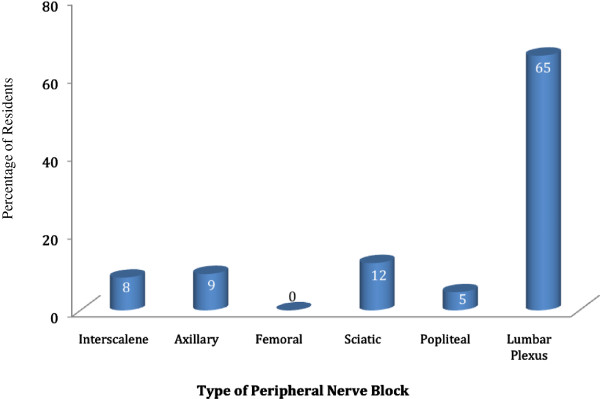
Percentage of residents who chose each block as the most difficult to perform.

**Figure 3:**
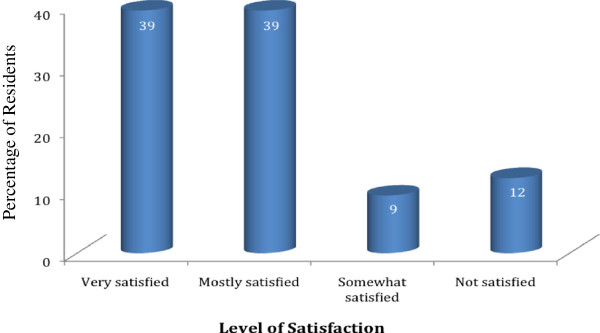
Level of resident satisfaction on peripheral nerve block education.

Out of 121 programs, 14 program directors agreed to forward the link to their senior residents. One program director specifically declined, and the rest could not be reached to confirm their program’s involvement. Thus, we estimate that approximately 250-300 senior anesthesia residents received the survey link from their respective program directors; an estimate of 300 was used to calculate the response rate. In total, 107 residents responded to the survey, yielding a response rate of 36%. The survey was programmed not to accept incomplete questionnaires. We received responses from all regions of the country with different sized programs (Table 1). The highest percentage of respondents was from the west (39%). Most respondents came from residency classes with greater than 20 residents per class (44%), with a similar percentage (41%) of respondents in classes of 11-20 residents, and the minority (15%) with class sizes of 1-10. Overall, 97 residents (91%) met the ACGME criteria for at least 40 peripheral nerve blocks. The majority of residents (74%) performed more than 60 PNBs (Table 1). Among the six types of PNBs surveyed, exposure to femoral, interscalene and popliteal blocks was high, as opposed to lumbar plexus blocks, which was low (Table 2). Overall, ultrasound-guided techniques were utilized more frequently than nerve stimulator-guided techniques for all of the PNBs except for lumbar plexus blocks (Figure 1). Most residents reported being “very comfortable” to “somewhat comfortable” performing all blocks, with the exception of lumbar plexus blocks, for which 64% were uncomfortable performing the block at all (Table 3). Indeed, the residents chose the lumber plexus block as the most difficult block to perform among the six PNBs (Figure 2). Residents were also asked about their comfort level in placing indwelling catheters. The most comfortable procedure for residents was the femoral nerve catheter, which the majority of residents (78%) felt “very comfortable” performing. In contrast, residents felt the least comfortable in placing lumber plexus catheters or axillary catheters (Table 3). Overall, 79% of residents indicated that they were “very satisfied” or “mostly satisfied” with the quality of the education they received during residency pertaining to peripheral nerve blocks. This left 9% of residents who were “somewhat satisfied” and another 12% who were “not satisfied” with the PNB education they received during residency (Figure 3). There was a significant correlation between the number of blocks performed and the confidence level of each resident, as well as the satisfaction with quality of teaching received during residency (Table 4). Residents were given four choices (more blocks, simulator training, didactic lectures and better equipment) and asked to rank the methods by which they felt they would gain the most confidence with PNBs. The majority of residents (90%) reported that “performing more blocks” would be the single most helpful method among the four suggested improvements. The second most helpful method selected for improving PNB education was “simulator training” (5%), followed by “didactic lectures” (3%), and “having better equipment” (2%).

**Table 1:** Demographics and clinical data (N=107)

	**N (%)**
Location of Residency Program*	
Northeast	18 (17)
Midwest	22 (21)
Southeast	11(10)
Southwest	14(13)
West	42 (39)
Number of Residents in Class	
11-5	5 (5)
6-10	11 (10)
11-15	26 (24)
16-20	18 (17)
More than 20	47 (44)
Total Number of PNBs Performed	
0-20	6 (6)
21-30	2 (2)
31-40	2 (2)
41-50	9 (8)
51-60	9 (8)
More than 60	79 (74)

*Northeast: New York, Pennsylvania, New Jersey, Maine, New Hampshire, Vermont, Massachusetts, Rhode Island, Connecticut. Midwest: Missouri, North Dakota, South Dakota, Nebraska, Kansas, Minnesota, Iowa, Wisconsin, Michigan, Illinois, Indiana, Ohio. Southeast: Delaware, Maryland, District of Columbia, Virginia, West Virginia, North Carolina, South Carolina, Georgia, Florida. Southwest: Kentucky, Tennessee, Mississippi, Alabama, Oklahoma, Texas, Arkansas, Louisiana. West: Washington, Oregon, California, Hawaii, Idaho, Montana, Wyoming, Nevada, Utah, Colorado, Arizona, New Mexico. PNBs = Peripheral Nerve Blocks.

**Table 2:** Type and number of peripheral nerve blocks performed (N=107)

	**Number of PNB, N (%)**		
Type	0-10	11-20	Over 20
Interscalene	36 (34)	27 (25)	44 (41)
Axillary	64 (60)	24 (22)	19 (18)
Femoral	18 (17)	26 (24)	63 (59)
Sciatic (Gluteal/Subgluteal)	61 (57)	23 (22)	23 (22)
Popliteal	34 (32)	40 (38)	33 (31)
Lumbar Plexus	90 (84)	7 (7)	10 (9)

PNB = peripheral nerve block.

**Table 3:** Confidence level with peripheral nerve blocks (N=107)

	**Confidence level with PNB, N (%)**				**Confidence level with placing indwelling catheter, N (%)**			
**Type**	**Very**	**Somewhat**	**Not**	**Mean (Median)**	**Very**	**Somewhat**	**Not**	**Mean (Median)**
Interscalene	73 (68)	24 (22)	10 (9)	1.41 (1)	47 (44)	34 (32)	26 (24)	1.80 (2)
Axillary	51 (48)	32 (30)	24 (22)	1.75 (2)	22 (21)	28 (26)	57 (53)	2.33 (3)
Femoral	93 (87)	7 (7)	7 (7)	1.20 (1)	83 (78)	16 (15)	8 (7)	1.30 (1)
Sciatic (Gluteal/ Subgluteal)	51 (48)	30 (28)	26 (24)	1.77 (2)	29 (27)	28 (26)	50 (47)	2.20 (2)
Popliteal	75 (70)	23 (21)	9 (8)	1.38 (1)	45 (42)	34 (32)	28 (26)	1.84 (2)
Lumbar Plexus	17 (16)	22 (21)	68 (64)	2.48 (3)	21 (20)	19 (18)	67 (63)	2.43 (3)

Mean and median of confidence level were calculated using the coding as follows: Very = 1; Somewhat = 2; Not = 3. PNB = peripheral nerve block.

**Table 4:** Correlations between number of blocks performed and confidence level (1), level of satisfaction (2)

	**1**		**2**	
**Type**	**Correlation**	**P-value**	**Correlation**	**P-value**
Interscalene	0.56	<0.0001	0.47	<0.0001
Axillary	0.66	<0.0001	0.28	0.004
Femoral	0.45	<0.0001	0.45	<0.0001
Sciatic (Gluteal/Subgluteal)	0.66	<0.0001	0.24	0.004
Popliteal	0.56	<0.0001	0.39	<0.0001
Lumbar Plexus	0.60	<0.0001	0.30	0.002

1. Spearman correlation between number of blocks performed and confidence.

2. Spearman correlation between number of blocks performed and level of satisfaction.

Positive correlation means that a higher number of blocks yields a higher level of satisfaction.

## Discussion

Reflecting the increasing popularity of PNBs, our study has shown that the number of PNBs performed during residency has significantly increased over the last decade. Smith et al. [4] reported that just over a decade ago, peripheral nerve blocks were substantially less utilized than neuraxial blocks. For example, the median number of femoral nerve blocks and interscalene blocks performed at the CA-3 level was 0 and 3, respectively. It is not surprising, then, that the majority of respondents in Smith’s survey reported feeling uncomfortable performing peripheral nerve blocks. In contrast, our study shows that 59% of respondents have performed over 20 femoral blocks and 41% of respondents have performed over 20 interscalene blocks. In our survey, the majority of residents report that they are “very comfortable” with femoral, popliteal and interscalene blocks. In addition, 48% of residents were also “very comfortable” with both axillary and sciatic blocks.

The current study illustrates that despite increasing experience with PNBs, deficiencies in confidence still exist. Although 91% of respondents met the ACGME criteria for 40 peripheral nerve blocks, 85% indicated they were either “not comfortable” or only “somewhat comfortable” with lumbar plexus blocks. Lumbar plexus blocks are considered by some to be more of a ‘central’ block and have resulted in major complications including cardiac arrest [7]. For both sciatic and axillary blocks, over half (52%) of respondents reported they were either “not comfortable” or only “somewhat comfortable” performing the blocks. Since the ACGME does not provide guidelines as to the specific types of blocks, it is possible that a resident may meet the requirement with a moderate to large number of one or two types of blocks, while being largely unfamiliar with many other types. Rosenblatt et al. [3] reported that the learning curve for interscalene blocks was steep. Only 50% of residents that had performed seven to nine previous blocks were able to perform the block autonomously, whereas 87.5% of residents who had performed fifteen prior blocks had autonomous success. Similarly, Konrad et al. [8] reported a 70% success rate after performance of 20 brachial plexus blocks. As Kopacz [9] concluded, our results suggest that 40 PNBs may be inadequate for the majority of residents to feel comfortable with a subset of PNBs commonly used in practice.

Due to the steep learning curve for PNBs, it seems reasonable that more exposure would be the most critical factor in improving resident education and confidence. Many programs have established ‘regional anesthesia’ rotations [10,11]. In addition, education at some institutions has been facilitated by standardized techniques described in training manuals that are given to residents prior to the beginning of their one- to two-month rotation [11]. It is thought that standardization of techniques may be helpful for the new learner who is unfamiliar with anatomical landmarks and ultrasound anatomy. Other programs have implemented a different model in which a third clinical anesthesia (CA-3) year resident is assigned to the preoperative area to perform regional anesthesia. Post-model analysis revealed a significant increase in the number of PNBs performed from an average of 80 to 350 [10]. A survey conducted in 2001 revealed that only 58% of programs had a specific PNB rotation or regional anesthesia rotation [12]. Implementation of a dedicated regional anesthesia rotation for residents should improve both the quantity and quality of education [9].

There are some potential limitations in the current study. Although we received responses from around the country and from programs of different sizes, the sampling strategy used in our survey may have resulted in a clustering of subjects. This sampling strategy and the inability to obtain information from non-responders may have led to limited representation across the region. This sampling strategy, the impersonal nature of the survey, and the lack of an associated reward in return for the respondent’s time may have attributed to the overall low response rate, estimated at 36%. In addition, we selected six PNBs to represent the spectrum of blocks utilized in both upper and lower extremity surgeries. We did not include other nerve blocks such as supraclavicular, trans-abdominal plane block, or obturator block. The addition of other nerve blocks would enable us to make a more comprehensive assessment of residents' experience and confidence level, but may have prohibitively lengthened the survey, thus further reducing the response rate. Lastly, the self-reported confidence level of residents is subjective and may vary from person to person. Confidence level does not necessarily correlate with the level of skill, or the success rate of the individual provider. That being said, our survey does provide some insight into the status of residents’ exposure and confidence level in performing nerve blocks.

## Conclusion

Resident experience with PNBs has grown immensely in the last decade as technology and techniques in this field have improved. Accordingly, the confidence level of graduating residents in performing PNBs has increased dramatically as more anesthesia providers become equipped to provide these services for their patients. Although most residents achieve the ACGME-mandated goal of 40 peripheral nerve blocks by the end of residency, some residents still do not feel confident in performing certain blocks by this deadline. Increasing resident exposure to peripheral nerve blockade, as well as incorporating simulation and more didactic lectures into resident education, would further increase the confidence level of residents in performing peripheral nerve blocks.

## Competing interests

The authors declare that they have no competing interests.

## Authors’ contributions

TSM participated in conducting the survey, and assisted in drafting the manuscript. EL performed the statistical analysis. SK designed and conducted the study and assisted in drafting the manuscript. All authors read and approved the final manuscript

## Pre-publication history

The pre-publication history for this paper can be accessed here:

http://www.biomedcentral.com/1471-2253/13/16/prepub
